# Dynamic response of the nonlocal strain-stress gradient in laminated polymer composites microtubes

**DOI:** 10.1038/s41598-020-61855-w

**Published:** 2020-03-27

**Authors:** Mohammad Amin Oyarhossein, As’ad Alizadeh, Mostafa Habibi, Mahmoud Makkiabadi, Mohsen Daman, Hamed Safarpour, Dong Won Jung

**Affiliations:** 1 Department of Civil Engineering, University of Aveiro, Aveiro, Portugal; 2 Department of Mechanical Engineering, Urmia University of Technology, Urmia, Iran; 3 Center of Excellence in Design, Robotics, and Automation, School of Mechanical Engineering, Sharif University of Technology, Tehran, Iran; 4 Department of Mechanical Engineering, Amirkabir University of Technology, Tehran, Iran; 5 Department of Mechanics, Imam Khomeini International University, Qazvin, Iran; 6 School of Mechanical Engineering, Jeju National University, Jeju, Jeju-do 690-756 South Korea

**Keywords:** Engineering, Mathematics and computing

## Abstract

This study presents the frequency analysis of a size-dependent laminated polymer composite microtube using a nonlocal strain-stress gradient (NSG) model. By applying energy methods (known as Hamilton’s principle), the motion equations of the laminated micro tube composites are developed. The thermodynamic equations of the laminated microtube are based on first-order shear deformation theory (FSDT), and a generalized differential quadrature method (GDQM) is employed to find the model for the natural frequencies. The results show that by considering C-F boundary conditions (BCs) and every even layers’ number in lower value of length scale parameter, the frequency of the structure drops by soaring this parameter. However, this matter is inverse in its higher value. Eventually, the ply angle’s influences, nonlocality as well as length scale element on the vibration of the laminated composite microstructure are investigated.

## Introduction

Reinforced laminated composites with graphene nanoplatelets (GPL) reinforcement are increasingly used in various applications due to its outstanding features, namely high tensile strength, high modulus, and lightweight^1–38^. Based on an experimental study, Rafiee *et al*.^39^ showed that the reinforced structures with GPL have better behaviors in comparison with them reinforced with multi-walled carbon nanotubes (MWCNT). Moreover, a considerable number of studies^13,23–25,27–33,40–50^ claimed that considering the GPL reinforcement in the epoxy matrix provides a significant improvement in the thermo-electro-mechanical properties^37,51,52^ and dynamic responses of the nanostructures^19,51–55^, based on this matter present work is a momentous field of study. Recently, the reinforcement is used in many applications such as sensor and actuator^56,57^. It is notable that when the size of a structure is changed from macro to nano/micro-scale the size-dependent effect should be considered using nonclassical theories^58–60^. Nonlocal strain-stress gradient (NSG) theory is one of those useful theories for estimating the mechanical behaviors of the micro/nano structures^58–60^. The wave responses of a beam with NSG theory is presented by Lim *et al*.^58^. Also, the size-dependent effect on the dynamic response of the nanobeams using a nonlocal theory is investigated in refs. ^59,60^. Besides, refs. ^27,34,61–79^ investigated the stability/instability analysis of the complex micro/nanostructures with the aid of analytical and numerical methods. In the scope of electro-mechanics of the shell with a piezo material, Shojaeefard *et al*.^73^ dealt with frequency analysis for different boundary conditions on a rotary cylindrical piezoelectric nanoshell surrounded by an elastic foundation. Also, they used the GDQ method for solving the problems. Electro-dynamical behavior of conical nanotubes applying moderately thin theory and a size-dependent theory has been studied by Dehkordi *et al*.^80^. Flex electric effects on the frequency of the nano-smart tube have been carried out in that paper. Arefi^81^ employed nonlocal elasticity theory and FSDT for investigation bending of double-curved size-dependent piezoelectric shells. Transverse loads and voltage are applied in that nano model surrounded by Pasternak and Winkler elastic foundations. They also examined the nonlocality, voltage, viscoelastic parameters on the electro-mechanic behaviors of the piezo nanostructure^82^. Razavi *et al*.^83^ published a paper about modeling a nanoshell made of functionally graded piezoelectric materials. They illustrated the impacts of dimensional parameters on the frequency of the mentioned nano model. Ninh and Bich^84^ demonstrated the nonlinear dynamic behavior of the electrically FG nano cylindrical shells in the thermal conditions. An FG shell reinforced with a carbon nanotube is taken into account in the inner and outer surfaces surrounded by piezo layers. Fang *et al*.^85^ engaged with thick theory and electro-mechanic model to study the nonlinear frequency of a size-dependent shell surrounded by a piezo layer. They studied the frequency curves of the nanoshell. Eftekhari *et al*.^86^ presented the dynamics of an FG cylindrical shell reinforced with carbon nanotube and the structure surrounded by PIAC in an orthotropic elastic medium and thermal site. They in this work applied an exact method along with DQ solution to figure out the equations and impacts of the electromagnetic field and a wide range of patterns of CNT ratio on dynamics of the system is presented. Vinyas^87^ encountered with FE modeling for frequency analysis of a plate which this structure has an MEE property. He considered moderately thick theory for modeling the problem. He emphasized that CNT pattern and volume of the reinforcement have a significant impact on the free vibration of the structure. Zhu *et al*.^88^ did a study on the free vibration of a PIAC nano cylindrical shell, and by employing the perturbation method, they solved the governing equations. They investigated the impact of surface energy on the dynamics of the nano smart structure. Fan *et al*.^89^ researched dynamics of a conic small scale structure. A couple of piezoelectric layers surrounded outer and inner layers of a conical CNTRC. It should be noted that this kind of structure can be used in the complex smart structures such as^37,51,52^. An intelligent controller equipped with a fast fault diagnosis method not only can guarantee the stability of a dynamic system but also it can predict or diagnose any fault in any complicated system^90^. For the first time, the presented study investigates the vibration analysis of a laminated composite microtube taking into consideration NSGT and exact values of nonlocalities and length scale parameters. The dynamic equations of the laminated microtube are based on FSDT and GDQM is implemented to solve these equations and obtain the natural frequency of the current model. Eventually, the current study has been made into the influences of the different types of the laminated parameters on the mechanical stability of the laminated composite microstructure employing continuum mechanics model.

## Mathematic Model

**Figure 1:**
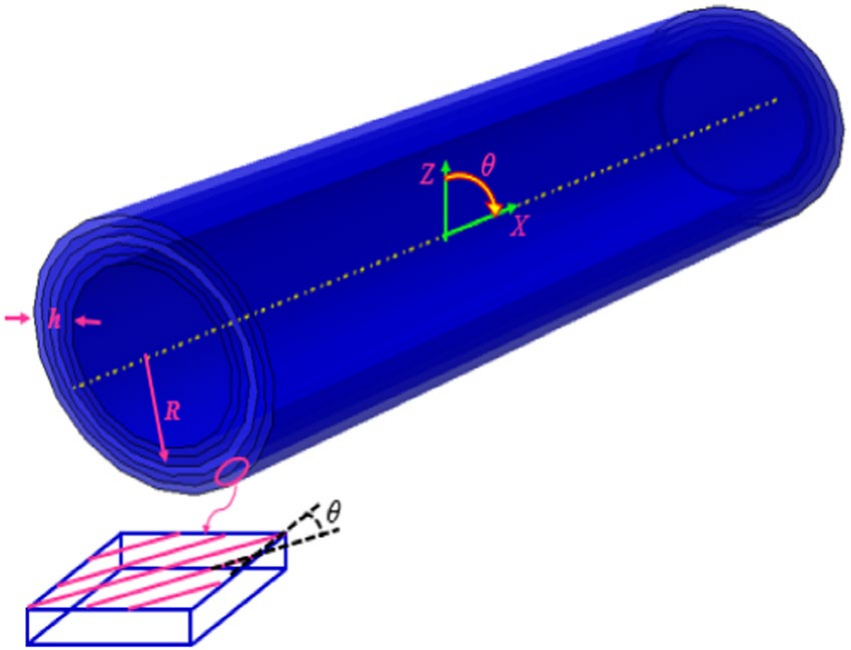
The geometry of a laminated composite microtube.

In Fig. 1, a laminated composite microtube with consideration of thermal effects is sketched, where *R* is the radius of the tube’s middle surface and *h* is the thickness of the microtube. Also, $\bar{\theta }$ is the ply angle of each layer. The material of the microstructure is considered as a laminated composite.

### NSG model

The fundamental equation can be expressed as follows due to the NSG model^35,91–93^:

1 $$(1-{\mu }^{2}{\nabla }^{2}){t}_{ij}={C}_{ijck}(1-{l}^{2}{\nabla }^{2}){\varepsilon }_{ck}$$

where, ${\nabla }^{2}={\partial }^{2}/\partial {x}^{2}+{\partial }^{2}/{R}^{2}\partial {\theta }^{2}$, ${t}_{ij}$, ${C}_{ijck}$, and ${\varepsilon }_{ck}$ respectively are the NSG stress, elasticity tensors, and strain. The tensor of NSG stress can be defined as follows^35^:

2 $${t}_{ij}={\sigma }_{ij}-\nabla {\sigma }_{ij}^{(1)}$$

here ${\sigma }_{ij}$ and ${\sigma }_{ij}^{(1)}$ presents the components of primary and micro size stresses, respectively. The *l* and *µ* are constant values standing for the higher-order strain gradient stress and non-invariant influence. Recent experimental researches also demonstrated the calibrated values of the size-dependent factors. The strain tensor could be written as:

3 $${\varepsilon }_{ij}=\frac{1}{2}({u}_{i,j}+{u}_{j,i})$$

where, ${u}_{i}$ stands for the elements of the displacement vector. Due to the Eq. (2), the relation between stress and strain of the mentioned structure would be presented as^94^:

4 $$\begin{array}{c}(1-{\mu }^{2}{\nabla }^{2})[\begin{array}{c}{t}_{xx}\\ {t}_{\theta \theta }\\ {t}_{x\theta }\end{array}]=(1-{l}^{2}{\nabla }^{2}){[\begin{array}{ccc}{C}_{11} & {C}_{12} & 0\\ {C}_{12} & {C}_{22} & 0\\ 0 & 0 & {C}_{66}\end{array}]}^{(L)}[\begin{array}{c}{\varepsilon }_{xx}\\ {\varepsilon }_{\theta \theta }\\ {\varepsilon }_{x\theta }\end{array}],\\ (1-{\mu }^{2}{\nabla }^{2})[\begin{array}{c}{t}_{\theta z}\\ {t}_{xz}\end{array}]=(1-{l}^{2}{\nabla }^{2}){[\begin{array}{cc}\begin{array}{c}{C}_{44}\\ 0\end{array} & \begin{array}{c}0\\ {C}_{55}\end{array}\end{array}]}^{(L)}[\begin{array}{c}{\varepsilon }_{\theta z}\\ {\varepsilon }_{xz}\end{array}]\end{array}$$

Equation (4) defines temperature changes as well as thermal expansion as $\Delta T$ and *α*, respectively. In the case of laminated composites, the elements of the tensor of elasticity are defined as the orthotropic material’s lessened elastic constants of the *L*th layer, and the next equations express the mentioned relations^94^:

5 $$\begin{array}{c}{C}_{11}={Q}_{11}{\cos }^{4}\bar{\theta }+2({Q}_{12}+2{Q}_{44}){\sin }^{2}\bar{\theta }{\cos }^{2}\bar{\theta }+{Q}_{22}{\sin }^{4}\bar{\theta }\\ {C}_{12}=({Q}_{11}+{Q}_{22}-4{Q}_{44}){\sin }^{2}\bar{\theta }{\cos }^{2}\bar{\theta }+{Q}_{12}({\sin }^{4}\bar{\theta }+{\cos }^{4}\bar{\theta })\\ {C}_{22}={Q}_{11}{\sin }^{4}\bar{\theta }+2({Q}_{12}+2{Q}_{44}){\sin }^{2}\bar{\theta }{\cos }^{2}\bar{\theta }+{Q}_{22}{\cos }^{4}\bar{\theta }\\ {C}_{44}={Q}_{44}{\cos }^{4}\bar{\theta }+{Q}_{55}{\sin }^{4}\bar{\theta }\\ {C}_{55}={Q}_{55}{\cos }^{4}\bar{\theta }+{Q}_{66}{\sin }^{4}\bar{\theta }\\ {C}_{66}=({Q}_{11}+{Q}_{22}-2{Q}_{12}){\sin }^{2}\bar{\theta }{\cos }^{2}\bar{\theta }+{Q}_{66}{({\cos }^{2}\bar{\theta }-{\sin }^{2}\bar{\theta })}^{2}\end{array}$$

The aforementioned equations express the relation between stress and strain components for the L*th* orthotropic lamina referred to as the lamina’s principal material axes *x*, $\,\theta$, and *z*. In Eq. (5), ${Q}_{ij}$ components are expressed by the following equations:

6 $$\begin{array}{ccc}{Q}_{11}=\frac{{E}_{1}}{1-{\nu }^{2}}, & {Q}_{12}=\frac{{\nu }_{12}{E}_{2}}{1-{\nu }^{2}}, & {Q}_{22}=\frac{{E}_{2}}{1-{\nu }^{2}}\\ {Q}_{66}={G}_{12}, & {Q}_{44}={G}_{23}, & {Q}_{55}={G}_{13}\end{array}$$

### Displacement field

FSDT enables us to define the displacement field of a laminated microtube as following equations:

7 $$\begin{array}{c}\,U({\rm{x}},\theta ,z,t)=u({\rm{x}},\theta ,z)+z{\psi }_{{\rm{x}}}({\rm{x}},\theta ,t)\\ V({\rm{x}},\theta ,z,t)=v({\rm{x}},\theta ,z)+z{\psi }_{\theta }({\rm{x}},\theta ,t)\\ W({\rm{x}},\theta ,z,t)=w({\rm{x}},\theta ,t)\end{array}$$

As well as that, $u(x,\theta ,t)$, $v(x,\theta ,t)$ along with $w(x,\theta ,t)$, respectively demonstrate the displacements of the neutral surface in *x* and $\theta$ axes. ${\psi }_{x}(x,\theta ,t)$ as well as ${\psi }_{\theta }(x,\theta ,t)$ illustrate the cross section rotations around $\theta$ and *x*- directions. By inserting Eq. (7) into Eq. (3), the strain tensor’s components can be obtained by the following equations:

8 $$\begin{array}{c}{\varepsilon }_{xx}=\frac{\partial u}{\partial x}+z\frac{\partial {\psi }_{x}}{\partial x}\\ {\varepsilon }_{\theta \theta }=\frac{1}{R}\frac{\partial v}{\partial \theta }+\frac{z}{R}\frac{\partial {\psi }_{\theta }}{\partial \theta }+\frac{w}{R}\\ {\varepsilon }_{xz}=\frac{1}{2}\left({\psi }_{x}+\frac{\partial w}{\partial x}\right)\\ {\varepsilon }_{x\theta }=\frac{1}{2}\left(\frac{1}{R}\frac{\partial u}{\partial \theta }+\frac{\partial v}{\partial x}\right)+\frac{z}{2}\left(\frac{1}{R}\frac{\partial {\psi }_{x}}{\partial \theta }+\frac{\partial {\psi }_{\theta }}{\partial x}\right)\\ {\varepsilon }_{\theta z}=\frac{1}{2}\left({\psi }_{\theta }+\frac{1}{R}\frac{\partial w}{\partial \theta }-\frac{v}{R}\right)\end{array}$$

### Governing equations and boundary conditions

The motion equations, along with the possible BCs related to the mentioned structure would be extracted applying energy methods (Hamilton principle) Based on FSDT and the NSG model by the following equation:

9 $${\int }_{{t}_{1}}^{{t}_{2}}(\delta K-\delta {\Pi }_{s})dt=0$$

here, *K* illustrates the kinetic energy, ${\Pi }_{s}$ defines strain energy and the work done by forces imposed can be shown as ${\Pi }_{w}$. For a usual micro tube exposed to the high level of temperature situation, it is suggested that the temperature distributes through its thickness.

Based on NSG model, Eq. (10) defines the strain energy^35^:

10 $$\delta K=\mathop{\int }\limits_{Z}\mathop{\iint }\limits_{A}\rho \left\{\begin{array}{c}\left(\frac{\partial u}{\partial t}+z\frac{\partial {\psi }_{x}}{\partial t}\right)\left(\frac{\partial }{\partial t}\delta u+z\frac{\partial }{\partial t}\delta {\psi }_{x}\right)+\\ \left(\frac{\partial v}{\partial t}+z\frac{\partial {\psi }_{\theta }}{\partial t}\right)\left(\frac{\partial }{\partial t}\delta v+z\frac{\partial }{\partial t}\delta {\psi }_{\theta }\right)+\left(\frac{\partial w}{\partial t}\right)\frac{\partial }{\partial t}\delta w\end{array}\right\}R\,dz\,dx\,d\theta $$

And also, the strain energy can be defined as the following equation due to the NSG model^35^:

11 $${\Pi }_{s}=\frac{1}{2}\mathop{\iiint }\limits_{V}({\sigma }_{ij}{\varepsilon }_{ij}+{\sigma }_{ij}^{(1)}\nabla {\varepsilon }_{ij})dV\Rightarrow \delta {\Pi }_{s}=\mathop{\iiint }\limits_{S}{t}_{ij}\delta {\varepsilon }_{ij}dV+\mathop{\iint }\limits_{A}{\sigma }_{ij}^{(1)}\delta {\varepsilon }_{ij}|\begin{array}{c}L\\ 0\end{array}dS$$

The components of the Eq. (11) and governing equations of the laminated microtube are given in the appendix.

## Solution Method

**Figure 2:**
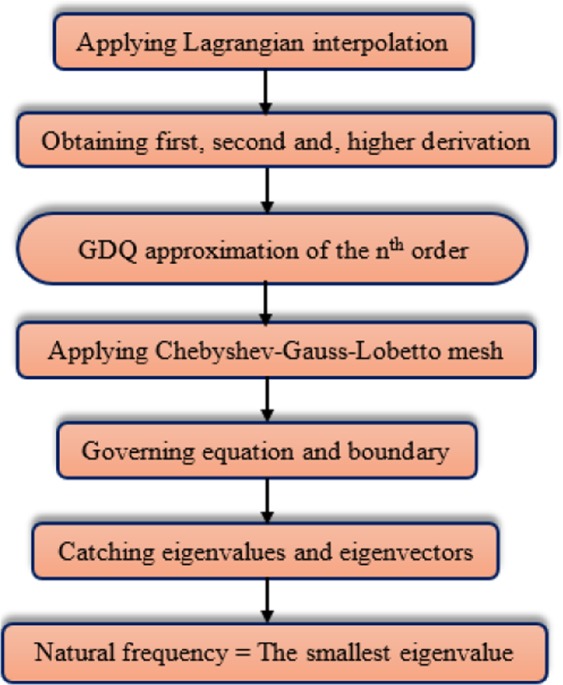
The flow chart of GDQM.

One of the best numerical methods which are well known for its accuracy and convergence is the Differential quadrature method (DQM)^95,96^. In this method, it is essential which the numbers of seed should be optimal^19,26,97–99^, which means that due to increasing the computational charge, too many seeds are not applicable, employing the few seeds, however, would lead to a negative impact on the accuracy of the results. At first, this method encounters its users with a limitation in which they could not use too many seeds owning to the algebraic weighting function. Shu^100,101^ improve the basic model of DQM with the aid of an explicit formula and decomposition technique so that he renamed the modified method to GDQ. GDQM is employed to find the solutions of governing equations beneath various boundary conditions. The flow chart of the aforementioned solution method is presented in Fig. 2.

With a view of this method estimated r-th defined by f(x) as follow:

12 $${\frac{{\partial }^{r}f(x)}{\partial {x}^{r}}|}_{x={x}_{p}}=\mathop{\sum }\limits_{j=1}^{n}{C}_{ij}^{(r)}f({x}_{i})$$

n and Cij called the number of seed and weighting coefficients in order which the second one computes as below:

13 $$\begin{array}{ll}{C}_{ij}^{(1)}=\frac{M({x}_{i})}{({x}_{i}-{x}_{j})M({x}_{j})} & i,j=1,2,\mathrm{..}.,n\,{\rm{and}}\,i\ne j\\ {C}_{ij}^{(1)}=-\,\mathop{\sum }\limits_{j=1,i\ne j}^{n}{C}_{ij}^{(1)} & i=j\end{array}$$

where,

14 $$M({x}_{i})=\,\mathop{\prod }\limits_{j=1,j\ne i}^{n}\,({x}_{i}-{x}_{j})$$

As well as these higher-order weight coefficients are as follows:

15 $$\begin{array}{ll}{C}_{ij}^{(r)}=r\left[{C}_{ij}^{(r-1)}{C}_{ij}^{(1)}-\frac{{C}_{ij}^{(r-1)}}{({x}_{i}-{x}_{j})}\right] & i,j=1,2,\mathrm{..}.,n,i\ne j\,{\rm{and}}\,2\le r\le n-1\\ {C}_{ii}^{(r)}=-\,\mathop{\sum }\limits_{j=1,i\ne j}^{n}{C}_{ij}^{(r)} & i,j=1,2,\mathrm{..}.,n\,{\rm{and}}\,1\le r\le n-1\end{array}$$

In the present research investigation, a seeds can be expressed as follows due to’ non-uniform set is chosen along x and $\theta$ excess:

16 $$\begin{array}{cc}{x}_{i}=\frac{L}{2}\left(1-\,\cos \left(\frac{(i-1)}{({N}_{i}-1)}\pi \right)\right) & i=1,2,3,\ldots ,{N}_{i}\end{array}$$

The freedom degrees can be taken into consideration as follows:

17 $$\begin{array}{c}u(x,\theta ,t)=U(x)\cos (n\theta ){e}^{i\omega t},\\ v(x,\theta ,t)=V(x)\sin (n\theta ){e}^{i\omega t},\\ w(x,\theta ,t)=W(x)\cos (n\theta ){e}^{i\omega t},\\ {\psi }_{x}(x,\theta ,t)={\Psi }_{x}(x)\cos (n\theta ){e}^{i\omega t},\\ {\psi }_{\theta }(x,\theta ,t)={\Psi }_{\theta }(x)\sin (n\theta ){e}^{i\omega t}.\end{array}$$

Reorganizing the quadrature analogs of boundary conditions along with field equations into the generalized eigenvalue problem’s fabric obtain:

18 $$\left\{\left[\begin{array}{cc}\left[{M}_{dd}\right] & \left[{M}_{db}\right]\\ \left[{M}_{bd}\right] & \left[{M}_{bb}\right]\end{array}\right]{\omega }^{2}+\left[\begin{array}{cc}\left[{K}_{dd}\right] & \left[{K}_{db}\right]\\ \left[{K}_{bd}\right] & \left[{K}_{bb}\right]\end{array}\right]\right\}\left\{\begin{array}{c}{\delta }_{d}\\ {\delta }_{b}\end{array}\right\}=0$$

where the subscripts *d* and *b* pertained to the grid points’ domain and boundary, respectively. As well as this, the displacement vector is shown by $\delta$. Equation (18), however, may be changed to a fundamental problem of eigenvalue:

19 $$\begin{array}{rcl}\left[{K}^{\ast }\right]\{{\delta }_{i}\} & = & \left({\omega }^{2}\right)\left[{M}^{\ast }\right]\{{\delta }_{i}\}\\ \left[{K}^{\ast }\right] & = & \left[{K}_{dd}-{K}_{db}{{K}_{bb}}^{-1}{K}_{bd}\right]\\ \left[{M}^{\ast }\right] & = & \left[{M}_{dd}-{M}_{db}{{K}_{bb}}^{-1}{K}_{bd}\right]\end{array}$$

As well as this, dimensionless natural frequency and dimensionless temperature difference are defined as bellow:

20 $$\varOmega =10\times \omega L\left(\sqrt{\frac{\rho }{E}}\right)$$

## Result and Discussion

In this paper, the laminated composite micro tube’s material properties are given in Table 1. The most prominent superiority of AS/3501 composite compared with conventional composites are their higher stiffness and strength as well as less density^102^.

**Table 1:** The material properties of AS/3501 graphite-epoxy layers^94^.

Material properties	E_1_	E_2_	G_12_	G_13_	G_23_	${{\boldsymbol{\alpha }}}_{{\bf{1}}}$	${{\boldsymbol{\alpha }}}_{{\bf{2}}}$	${\boldsymbol{\nu }}$
Values	140 GPa	10 GPa	7 GPa	7 GPa	7 GPa	$-0.3\times {10}^{-6}/K$	$28\times {10}^{-6}/K$	0.078

### Convergencey

**Figure 3:**
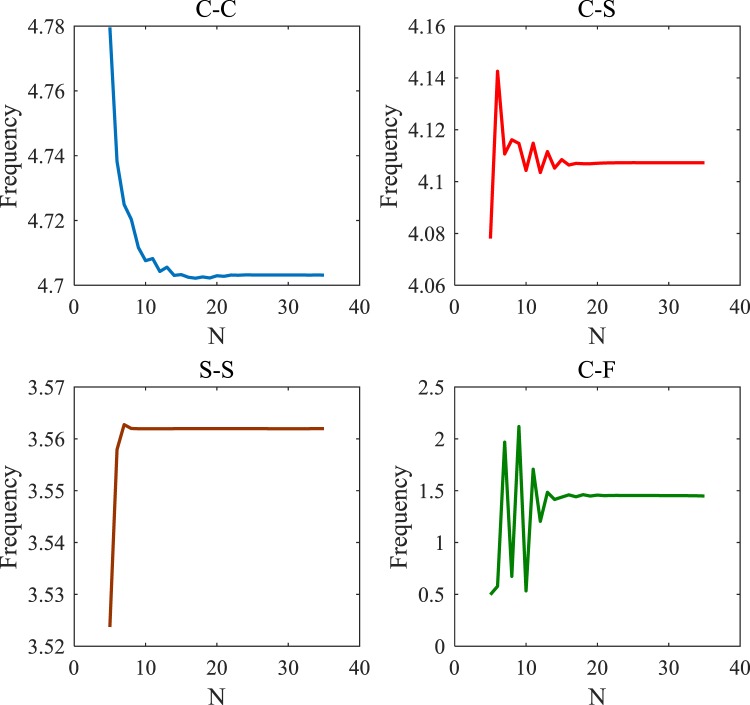
The grid point numbers’ effects on the convergency of the results for the nondimensional frequency of the laminated composites microtube for different boundary conditions when *L/R* = 10, *h/R* = 0.1, $\,\mu =l=0.1\,nm$.

Achieving a higher degree of results accuracy in the GDQ solution method is strongly dependent on adequate grid point numbers. The convergence has been conducted for a range of materials along with various boundary conditions (Clamped-Clamped (C-C), Clamped-Simply (C-S), Simply-Simply (S-S), and Clamped-Free (C-F)). At the same time, this would be observed that the stiffness of microtube under C-C boundary conditions is much more than the microtube under C-F boundary conditions leading to a lower nondimensional critical temperature. According to Fig. 3, twenty grid points are adequate for the convergence of the results presented.

### Validation

For validating the results presented in this study with other research papers, Table 2 evaluates outcomes for the micro tube’s nondimensional frequency and the outcomes presented by ref. ^103^, for different geometrical parameters. Besides, the results disclose that the decrease of nondimensional length scale element (*h/l*) may result in a drop in the natural frequency. The mentioned formulation, however, is validated by those available in the literature. Table 2 illustrates a decent agreement between the presented outcomes and reference.

**Table 2:** Evaluation of three vibrational modes of isotropic homogeneous microtube (various thickness values are considered).

h/R	*n*	ref. ^103^ (*l* = 0)	Present research (*l* = 0)	ref. ^103^ (*l* = h)	Present research (*l* = h)
0.02	1	0.1954	0.1954	0.1955	0.1954
	2	0.2532	0.2527	0.2575	0.2573
	3	0.2772	0.2758	0.3067	0.3062
0.05	1	0.1959	0.1954	0.1963	0.1958
	2	0.2623	0.2588	0.2869	0.2854
	3	0.3220	0.3140	0.4586	0.4545

### length scale Influences on the frequency of the laminated composite microstructure

**Figure 4:**
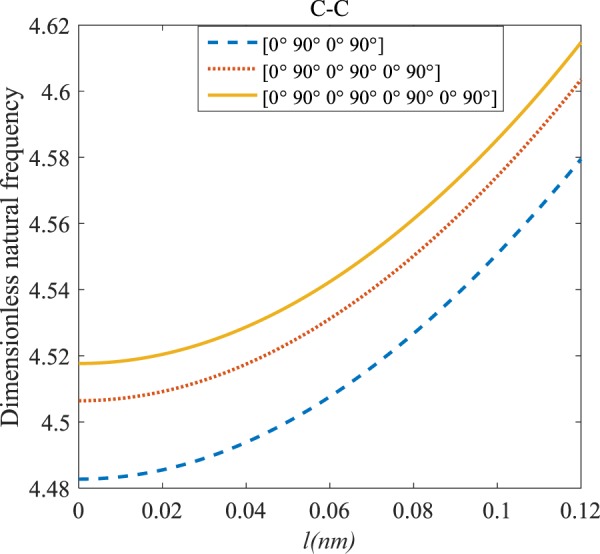
The effects of *l* and even layers number on the vibration of the structure under the boundary condition of C-C.

**Figure 5:**
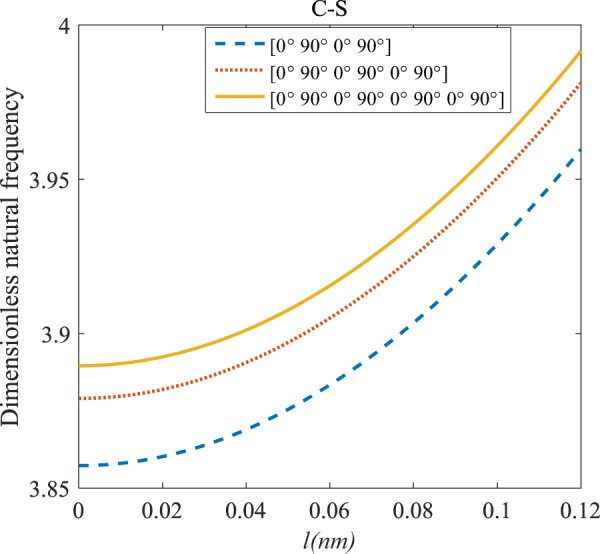
The effects of *l* and even layers number on the vibration of the structure under the boundary condition of C-S.

**Figure 6:**
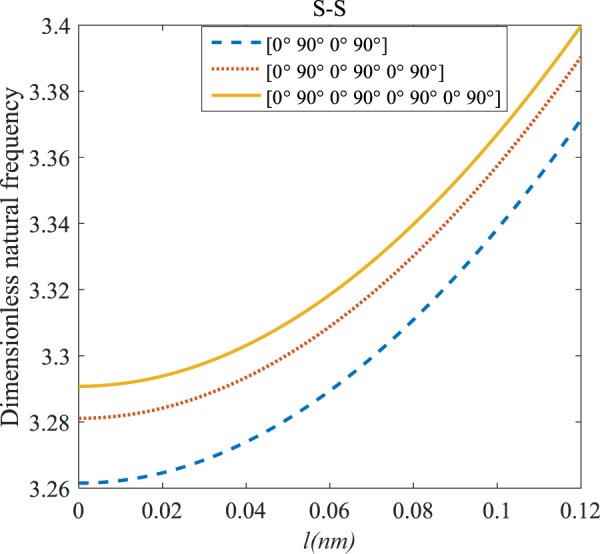
The effects of *l* and even layers number on the vibration of the structure under the boundary condition of S-S.

**Figure 7:**
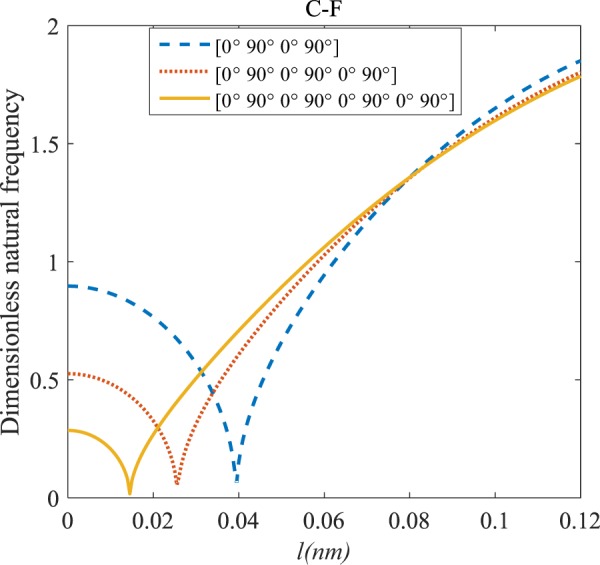
The effects of *l* and even layers’ number on the vibration of the structure under the boundary condition of C-F.

**Figure 8:**
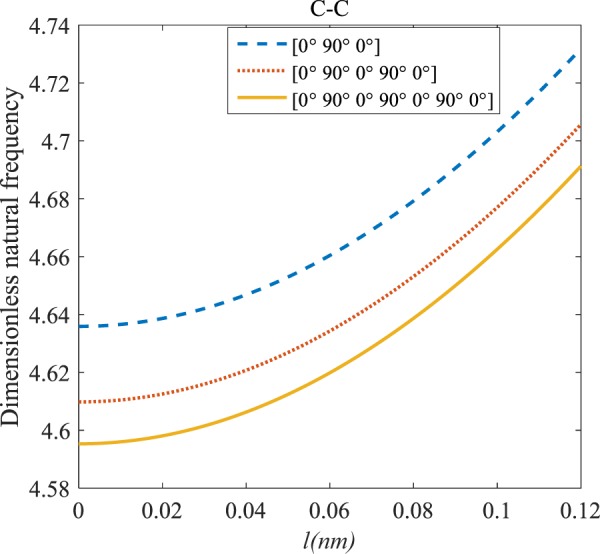
The effects of *l* and odd layers number on the vibration of the structure under the boundary condition of C-C.

**Figure 9:**
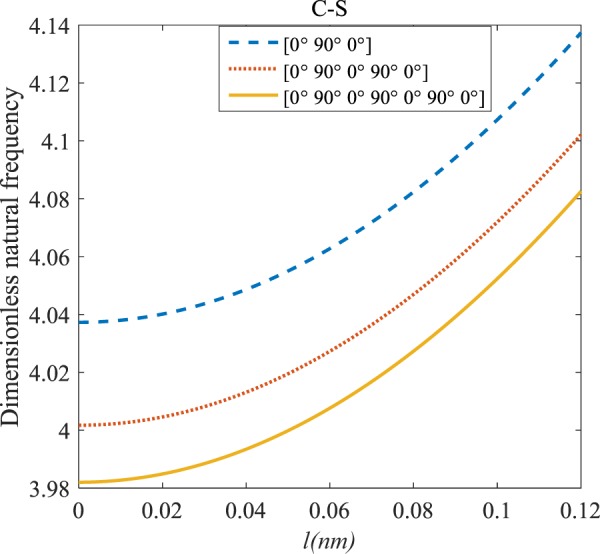
The effects of *l* and odd layers number on the vibration of the structure under the boundary condition of C-S.

**Figure 10:**
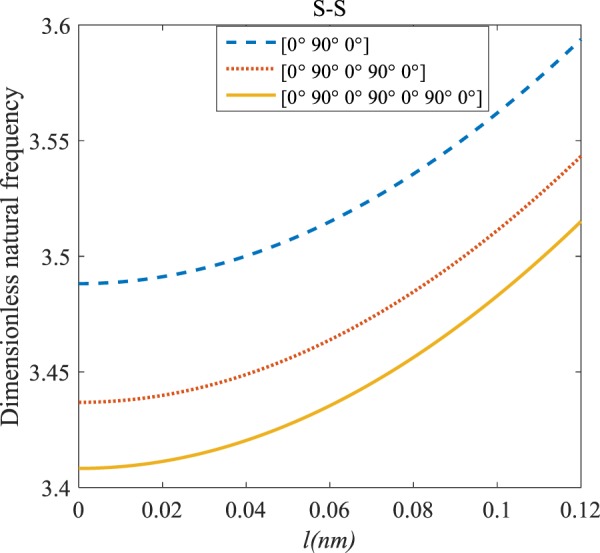
The effects of *l* and odd layers number on the vibration of the structure under the boundary condition of S-S.

**Figure 11:**
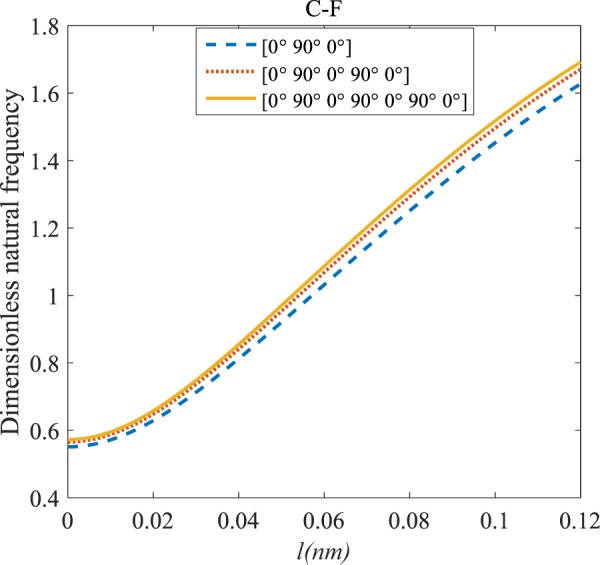
Effects of *l* and odd layers’ number on the vibration of the structure under boundary conditions of C-F.

Figures 4–11 illustrate the influence of the various angles of symmetric laminate, the number of layers and length scale element on the frequency for a range of boundary conditions. The proposed structure is a laminated composite microtube with *R* = 1 nm and *h* = *R*/10. The small scale factors are suggested to be *µ* = 0.55 nm, *l* = 0.35 nm in the relevant models^35^.

#### Even–layered laminates’ comparison

According to Figs. 4–7, for C-C, C-S as well as S-S boundary conditions, increasing the length scale parameter, the figures presented to demonstrate a similar behavior in the all mentioned cases. By rising the element of length scale, the frequency of the microstructure increases. These figures present that, by boosting the even layers’ number of the laminated composite, the frequency of the structure increases. Such increases are considerable for C-C boundary conditions and boost the stability of such structures. The difference between Figs. 4–6 are that the dimensionless frequency parameter of the C-C boundary condition is more than C-S and S-S boundary conditions. This is because, in the case of the C-C boundary condition, the microstructure stability would be enhanced. Also, a new result is presented in the boundary condition of C-F in Fig. 7. For this regard it may be observed, the effect of length scale factor on the frequency is much more changeable. Moreover, for every even layer number, in lower value of length scale factor $(0\le {l}^{forfourlayers}\le 0.0183,0\le {l}^{forsixlayers}\le 0.0278,0\le {l}^{foreightlayers}\le 0.04)$, by raising the value of length scale factor, the frequency of the structure drops but in higher value $({l}^{forfourlayers}\ge 0.0183,$ ${l}^{forsixlayers}\ge 0.0278,{l}^{foreightlayers}\ge 0.04)$ of length scale parameter, this matter is inverse. Besides, this figure shows that even layers’ number effect on the frequency, change in *l*=0.872 nm. So, for length scale parameter less than 0.872 nm, whenever the composite layers increase, the frequency increases as well, while for $l > 0.872nm$ the reverse is true.

#### Odd–layered laminates’ comparison

The dimensionless frequency respect to the length scale factor for various odd layers’ numbers of the laminated composite and S-S, C-S, C-C along with C-F boundary conditions are depicted in Figs. 8–11. It is observed that rising the length scale factor causes the frequency of the system to increase. It is clear from Figs. 8–11, because of increasing stiffness of structure with rising odd layers’ number, the variation of frequency with an increase of odd layers’ number decreases. As mentioned earlier, the dynamic stability can be enhanced if the length scale factor increases. This enhancement is more significant in the C-C boundary condition. The difference between these figures is that the effects of odd layers’ numbers on the vibration of the structure with C-F boundary conditions are much less than in comparison with other boundary conditions. For more comprehensive, the odd layers’ number indeed has a positive effect on the frequency of the microtube with C-F boundary condition, but this effect is minimal and can be ignored.

### Influences of the nonlocal parameter on the frequency of the laminated composite microstructure

Figures 12–19 demonstrate the impact of the angles’ different symmetric laminate, layers number as well as nonlocality on the vibration for different boundary conditions.

**Figure 12:**
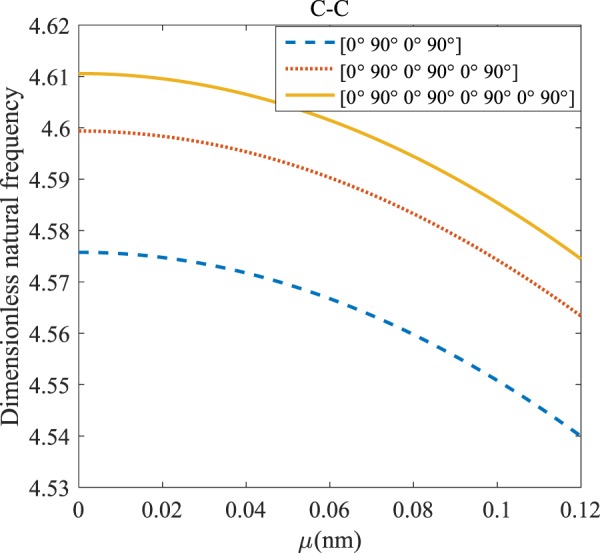
The effects of *μ* and even layers number on the vibration of the structure under the boundary condition of C-C.

**Figure 13:**
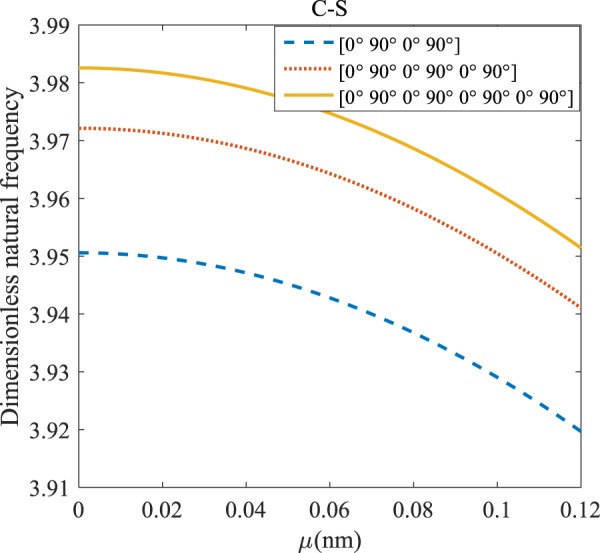
The effects of *μ* and even layers number on the vibration of the structure under the boundary condition of C-S.

**Figure 14:**
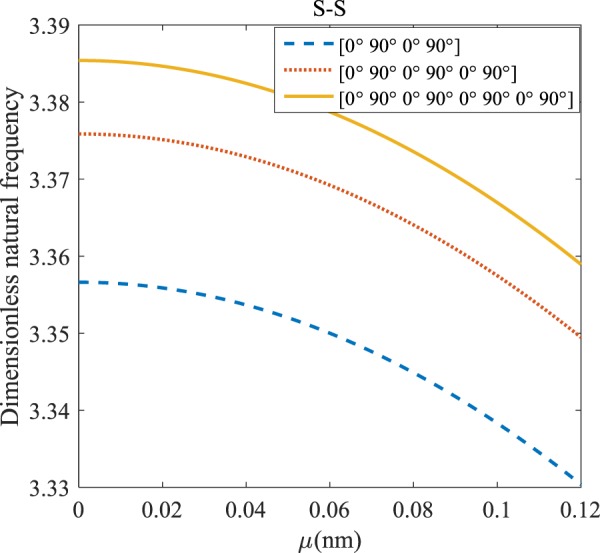
The effects of *μ* and even layers number on the vibration of the structure under the boundary condition of S-S.

**Figure 15:**
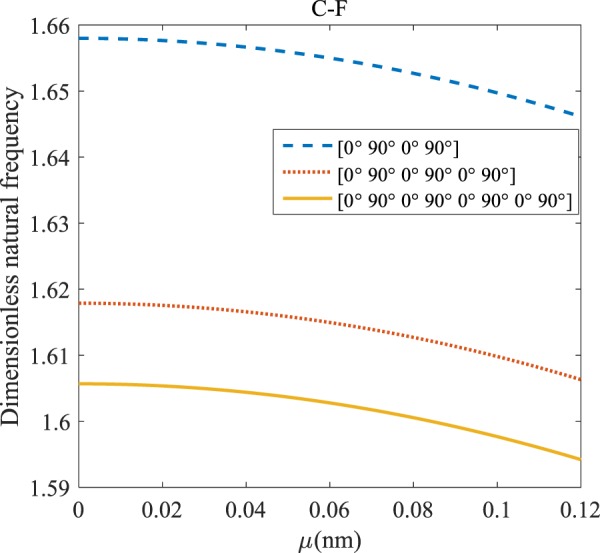
Effects of *μ* and even layers number on the vibration of the structure under the boundary condition of C-F.

**Figure 16:**
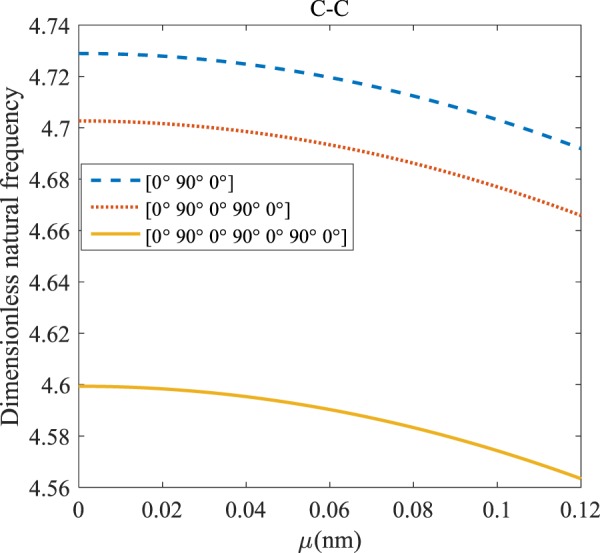
The effects of *μ* and odd layers number on the vibration of the structure under C-C boundary conditions.

**Figure 17:**
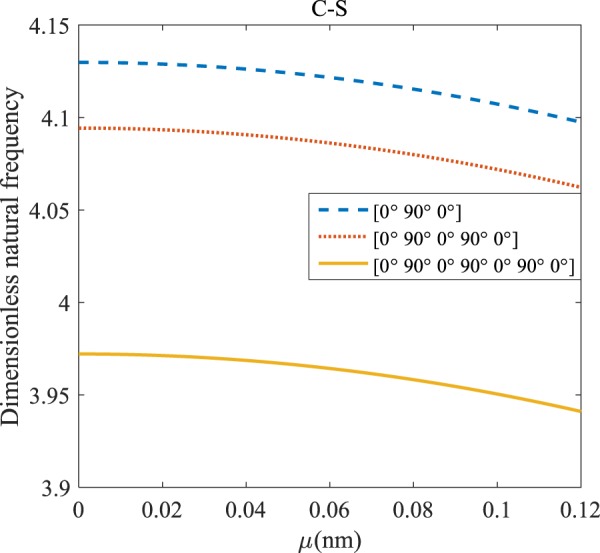
The effects of *μ* and odd layers number on the vibration of the structure under C-S boundary conditions.

**Figure 18:**
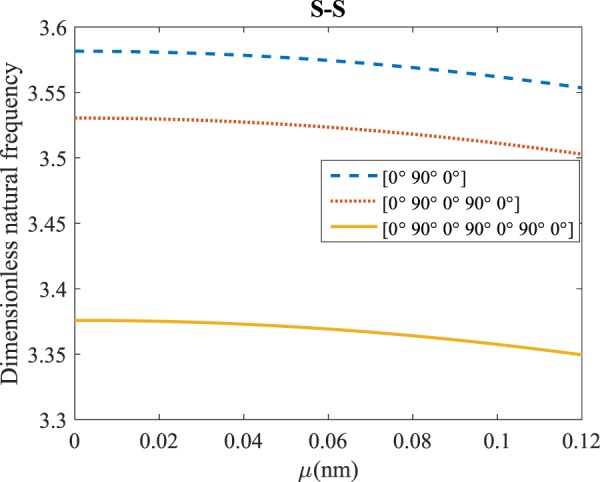
The effects of *μ* and odd layers number on the vibration of the structure under S-S boundary conditions.

**Figure 19:**
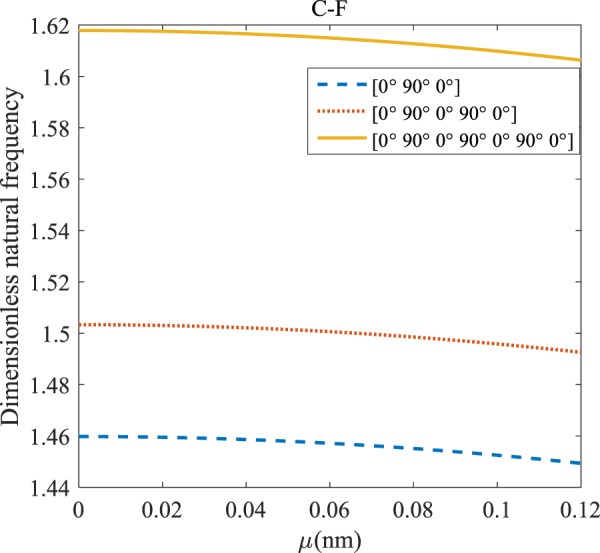
The effects of *μ* and odd layers number on the vibration of the structure under C-F boundary conditions.

#### Even–layered laminates’ comparison

Regarding Figs. 12–15, for C-C, C-S, S-S, and C-F boundary conditions, increasing the nonlocal parameter, all figures demonstrate similar mechanical behavior. By rising the nonlocal parameter, the frequency of the micro-scaled structure drops. These figures present that, by boosting the even layers’ number of the laminated composite, the frequency of the microstructure increases. The mentioned increment is considerable for C-C boundary conditions and enhances the stability of the structure. For more comprehensive, increasing even layers’ numbers indeed has a positive effect on the frequency but, nonlocality has an inverse influence on the frequency. The difference between Figs. 12–15 is that the dimensionless fundamental vibration mode of the C-C boundary condition is more than other boundary conditions. This means the C-C boundary condition improves the structure stability. As mentioned earlier, increasing layers’ numbers have a direct effect on the frequency so, and this positive point is much more significant than when the boundary condition is C-F. As a new result for literature, increasing even layers’ numbers play a prominent role in the stability of the C-F laminated composite microtube.

#### Odd–layered laminates’ comparison

The dimensionless frequency versus the nonlocal parameter for different odd layers number of the laminated composite and S-S, C-S, C-C, and C-F boundary conditions are depicted in Figs. 16–19. It can be illustrated that increasing the nonlocal parameter causes the frequency of the system to decreases. It is clear from Figs. 16–19, because of increasing stiffness of structure with rising odd layers’ number, the variation of frequency with increasing of odd layers’ number increases. As mentioned earlier, by increasing the nonlocal parameter, the dynamic stability is boosted. This enhancement is more significant in the C-C boundary condition. The difference between these figures is that the effects of odd layers’ numbers on the frequency of the structure with C-F boundary conditions are much more than in comparison with other boundary conditions.

## Conclusion

The present research work investigated the stability of a small-scaled laminated composite microtube using the NSG model. The governing motion equations pertained to the laminated composite microtube have been obtained by employing energy methods, and GDQM enabled us to solve the obtained equations. The current investigation evaluated dynamic stability analysis of a laminated composite microtube considering continuum mechanics for the first time. Ultimately, by employing the mentioned continuum theory, this study has been made into the impact of the various kinds of laminated composite microtube parameters on the vibrational characteristics of the microstructure. The most prominent results obtained in the current paper can be found as follows:

1. For C-F boundary condition and every even layers’ number, in the lower values of length scale parameter, as this factor increases, the fundamental frequency of the structure decreases but in higher values of length scale factor this matter becomes inverse.
2. For the C-F boundary condition and even layers’ number, the impact of the length scale factor on the natural frequency is more changeable.
3. The more the length scale values and the layers’ number increase, the more structure’s frequency becomes for C-C, C-S as well as S-S boundary conditions and every even and odd layers’ number.
4. The odd layers’ number has a positive effect on the frequency of the microtube with C-F boundary condition, but this effect is minimal and can be ignored.

## Appendix

The strain energy variation can be derived as the following equations:

1 $$\begin{array}{ccc}\delta {\Pi }_{s} & = & {\iint }_{A}\{\begin{array}{c}{N}_{xx}\frac{\partial }{\partial x}\delta u+{M}_{xx}\frac{\partial }{\partial x}\delta {\psi }_{x}+{N}_{\theta \theta }(\frac{1}{R}\frac{\partial }{\partial \theta }\delta v+\frac{1}{R}\delta w)\\ +\frac{1}{R}{M}_{\theta \theta }\frac{\partial }{\partial \theta }\delta {\psi }_{\theta }+{Q}_{xz}(\delta {\psi }_{x}+\frac{\partial }{\partial x}\delta w)+{N}_{x\theta }(\frac{1}{R}\frac{\partial }{\partial \theta }\delta u+\frac{\partial }{\partial x}\delta v)\\ +{M}_{x\theta }(\frac{1}{R}\frac{\partial }{\partial \theta }\delta {\psi }_{x}+\frac{\partial }{\partial x}\delta {\psi }_{\theta })+{Q}_{z\theta }(\delta {\psi }_{\theta }+\frac{1}{R}\frac{\partial }{\partial \theta }\delta w-\frac{1}{R}\delta v)\end{array}\}Rdxd\theta \\  &  & +\int \{\begin{array}{c}{N}_{xx}^{(1)}\frac{\partial }{\partial x}\delta u+{M}_{xx}^{(1)}\frac{\partial }{\partial x}\delta {\psi }_{x}+{N}_{\theta \theta }^{(1)}(\frac{1}{R}\frac{\partial }{\partial \theta }\delta v+\frac{1}{R}\delta w)\\ +\frac{1}{R}{M}_{\theta \theta }^{(1)}\frac{\partial }{\partial \theta }\delta {\psi }_{\theta }+{Q}_{xz}^{(1)}(\delta {\psi }_{x}+\frac{\partial }{\partial x}\delta w)+{N}_{x\theta }^{(1)}(\frac{1}{R}\frac{\partial }{\partial \theta }\delta u+\frac{\partial }{\partial x}\delta v)\\ +{M}_{x\theta }^{(1)}(\frac{1}{R}\frac{\partial }{\partial \theta }\delta {\psi }_{x}+\frac{\partial }{\partial x}\delta {\psi }_{\theta })+{Q}_{\theta z}^{(1)}(\delta {\psi }_{\theta }+\frac{1}{R}\frac{\partial }{\partial \theta }\delta w-\frac{1}{R}\delta v)\end{array}\}|\begin{array}{c}L\\ 0\end{array}Rd\theta \end{array}$$

where the momentum and force resultants are:

2 $$\begin{array}{c}\{\begin{array}{c}{N}_{xx},{N}_{\theta \theta },{N}_{x\theta }\\ {N}_{xx}^{(1)},{N}_{\theta \theta }^{(1)},{N}_{x\theta }^{(1)}\end{array}\}={\int }_{-h/2}^{h/2}\{\begin{array}{c}{t}_{xx},{t}_{\theta \theta },{t}_{x\theta }\\ {\sigma }_{xx}^{(1)},{\sigma }_{\theta \theta }^{(1)},{\sigma }_{x\theta }^{(1)}\end{array}\}dz\\ \{\begin{array}{c}{M}_{xx},{M}_{\theta \theta },{M}_{x\theta }\\ {M}_{xx}^{(1)},{M}_{\theta \theta }^{(1)},{M}_{x\theta }^{(1)}\end{array}\}={\int }_{-h/2}^{h/2}\{\begin{array}{c}{t}_{xx},{t}_{\theta \theta },{t}_{x\theta }\\ {\sigma }_{xx}^{(1)},{\sigma }_{\theta \theta }^{(1)},{\sigma }_{x\theta }^{(1)}\end{array}\}zdz\\ \{\begin{array}{c}{Q}_{xz},{Q}_{\theta z}\\ {Q}_{xz}^{(1)},{Q}_{\theta z}^{(1)}\end{array}\}={\int }_{-h/2}^{h/2}{K}_{s}\{\begin{array}{c}{t}_{xz},{t}_{\theta z}\\ {\sigma }_{xz}^{(1)},{\sigma }_{\theta z}^{(1)}\end{array}\}dz\end{array}$$

Governing motion equations for a microtube due to the FSDT as well as NSG model are presented inserting Eqs. (10), (11) into Eq. (9) and integrating as follows:

3 $$\begin{array}{cc} & \delta u:{A}_{11}(\frac{{\partial }^{2}u}{\partial {x}^{2}}+{l}^{2}\frac{{\partial }^{4}u}{\partial {x}^{4}}-\frac{{l}^{2}}{{R}^{2}}\frac{{\partial }^{4}u}{\partial {x}^{2}\partial {\theta }^{2}})+{B}_{11}(\frac{{\partial }^{2}{\psi }_{x}}{\partial {x}^{2}}+{l}^{2}\frac{{\partial }^{4}{\psi }_{x}}{\partial {x}^{4}}-\frac{{l}^{2}}{{R}^{2}}\frac{{\partial }^{4}{\psi }_{x}}{\partial {x}^{2}\partial {\theta }^{2}})\\  & +{A}_{12}(\frac{1}{R}\frac{{\partial }^{2}v}{\partial x\partial \theta }+\frac{1}{R}\frac{\partial w}{\partial x}-\frac{{l}^{2}}{{R}^{2}}\frac{{\partial }^{4}v}{\partial {x}^{3}\partial \theta }-\frac{{l}^{2}}{{R}^{2}}\frac{{\partial }^{3}w}{\partial {x}^{3}}-\frac{{l}^{2}}{{R}^{3}}\frac{{\partial }^{4}v}{\partial x\partial {\theta }^{3}}-\frac{{l}^{2}}{{R}^{3}}\frac{{\partial }^{3}w}{\partial x\partial {\theta }^{2}})\\  & +{B}_{12}(\frac{1}{R}\frac{{\partial }^{2}{\psi }_{\theta }}{\partial x\partial \theta }-\frac{{l}^{2}}{R}\frac{{\partial }^{4}{\psi }_{\theta }}{\partial {x}^{3}\partial \theta }-\frac{{l}^{2}}{{R}^{3}}\frac{{\partial }^{4}{\psi }_{\theta }}{\partial x\partial {\theta }^{3}})-{N}_{h}(\frac{1}{R}\frac{{\partial }^{2}v}{\partial x\partial \theta }-\frac{1}{{R}^{2}}\frac{{\partial }^{2}u}{\partial {\theta }^{2}})\\  & +\frac{{A}_{66}}{R}(\frac{1}{R}\frac{{\partial }^{2}u}{\partial {\theta }^{2}}+\frac{{\partial }^{2}v}{\partial x\partial \theta }-\frac{{l}^{2}}{R}\frac{{\partial }^{4}u}{\partial {x}^{2}\partial {\theta }^{2}}-{l}^{2}\frac{{\partial }^{4}v}{\partial {x}^{3}\partial \theta }-\frac{{l}^{2}}{{R}^{3}}\frac{{\partial }^{4}u}{\partial {\theta }^{4}}-\frac{{l}^{2}}{{R}^{2}}\frac{{\partial }^{4}v}{\partial x\partial {\theta }^{3}})\\  & +\frac{{B}_{66}}{R}(\frac{1}{R}\frac{{\partial }^{2}{\psi }_{x}}{\partial {\theta }^{2}}+\frac{{\partial }^{2}{\psi }_{\theta }}{\partial x\partial \theta }-\frac{{l}^{2}}{R}\frac{{\partial }^{4}{\psi }_{x}}{\partial {x}^{2}\partial {\theta }^{2}}-{l}^{2}\frac{{\partial }^{4}{\psi }_{\theta }}{\partial {x}^{3}\partial \theta }-\frac{{l}^{2}}{{R}^{3}}\frac{{\partial }^{4}{\psi }_{x}}{\partial {\theta }^{4}}-\frac{{l}^{2}}{{R}^{2}}\frac{{\partial }^{4}{\psi }_{\theta }}{\partial x\partial {\theta }^{3}})\\ = & (1-{\mu }^{2}{\nabla }^{2})({I}_{0}\frac{{\partial }^{2}u}{\partial {t}^{2}}+{I}_{1}\frac{{\partial }^{2}{\psi }_{x}}{\partial {t}^{2}})\end{array}$$

4 $$\begin{array}{c}\delta v:\frac{{A}_{12}}{R}(\frac{{\partial }^{2}u}{\partial x\partial \theta }+{l}^{2}\frac{{\partial }^{4}u}{\partial {x}^{3}\partial \theta }-\frac{{l}^{2}}{{R}^{2}}\frac{{\partial }^{4}u}{\partial x\partial {\theta }^{3}})+\frac{{B}_{12}}{R}(\frac{{\partial }^{2}{\psi }_{x}}{\partial x\partial \theta }+{l}^{2}\frac{{\partial }^{4}{\psi }_{x}}{\partial {x}^{3}\partial \theta }-\frac{{l}^{2}}{{R}^{2}}\frac{{\partial }^{4}{\psi }_{x}}{\partial x\partial {\theta }^{3}})\\ +\frac{{A}_{22}}{R}(\frac{1}{R}\frac{{\partial }^{2}v}{\partial {\theta }^{2}}+\frac{1}{R}\frac{\partial w}{\partial \theta }-\frac{{l}^{2}}{{R}^{2}}\frac{{\partial }^{4}v}{\partial {x}^{2}\partial {\theta }^{2}}-\frac{{l}^{2}}{{R}^{2}}\frac{{\partial }^{3}w}{\partial {x}^{2}\partial \theta }-\frac{{l}^{2}}{{R}^{3}}\frac{{\partial }^{4}v}{\partial {\theta }^{4}}-\frac{{l}^{2}}{{R}^{3}}\frac{{\partial }^{3}w}{\partial {\theta }^{3}})\\ +\frac{{B}_{22}}{R}(\frac{1}{R}\frac{{\partial }^{2}{\psi }_{\theta }}{\partial {\theta }^{2}}+\frac{{l}^{2}}{R}\frac{{\partial }^{4}{\psi }_{\theta }}{\partial {x}^{2}\partial {\theta }^{2}}-\frac{{l}^{2}}{{R}^{3}}\frac{{\partial }^{4}{\psi }_{\theta }}{\partial {\theta }^{4}})\\ +{A}_{66}(\frac{1}{R}\frac{{\partial }^{2}u}{\partial x\partial \theta }+\frac{{\partial }^{2}v}{\partial {x}^{2}}-\frac{{l}^{2}}{R}\frac{{\partial }^{4}u}{\partial {x}^{3}\partial \theta }-{l}^{2}\frac{{\partial }^{4}v}{\partial {x}^{4}}-\frac{{l}^{2}}{{R}^{3}}\frac{{\partial }^{4}u}{\partial x\partial {\theta }^{3}}-\frac{{l}^{2}}{{R}^{2}}\frac{{\partial }^{4}v}{\partial {x}^{2}\partial {\theta }^{2}})\\ +{B}_{66}(\frac{1}{R}\frac{{\partial }^{2}{\psi }_{x}}{\partial x\partial \theta }+\frac{{\partial }^{2}{\psi }_{\theta }}{\partial {x}^{2}}-\frac{{l}^{2}}{R}\frac{{\partial }^{4}{\psi }_{x}}{\partial {x}^{3}\partial \theta }-{l}^{2}\frac{{\partial }^{4}{\psi }_{\theta }}{\partial {x}^{4}}-\frac{{l}^{2}}{{R}^{3}}\frac{{\partial }^{4}{\psi }_{x}}{\partial x\partial {\theta }^{3}}-\frac{{l}^{2}}{{R}^{2}}\frac{{\partial }^{4}{\psi }_{\theta }}{\partial {x}^{2}\partial {\theta }^{2}})\\ +\frac{{K}_{s}{A}_{44}}{R}({\psi }_{\theta }+\frac{1}{R}\frac{\partial w}{\partial \theta }-\frac{v}{R}-{l}^{2}\frac{{\partial }^{2}{\psi }_{\theta }}{\partial {x}^{2}}-\frac{{l}^{2}}{{R}^{2}}\frac{{\partial }^{3}w}{\partial {x}^{2}\partial \theta }+\frac{{l}^{2}}{{R}^{2}}\frac{{\partial }^{2}v}{\partial {x}^{2}}\\ -\frac{{l}^{2}}{{R}^{2}}\frac{{\partial }^{2}{\psi }_{\theta }}{\partial {\theta }^{2}}-\frac{{l}^{2}}{{R}^{3}}\frac{{\partial }^{3}w}{\partial {\theta }^{3}}+\frac{{l}^{2}}{{R}^{3}}\frac{{\partial }^{2}v}{\partial {\theta }^{2}})=(1-{\mu }^{2}{\nabla }^{2})\left({I}_{0}\left[\frac{{\partial }^{2}v}{\partial {t}^{2}}\,\right]+{I}_{1}\left[\frac{{\partial }^{2}{\psi }_{\theta }}{\partial {t}^{2}}\right]\right)\end{array}$$

5 $$\begin{array}{c}\delta w:\frac{{A}_{12}}{R}(\frac{\partial u}{\partial x}+{l}^{2}\frac{{\partial }^{3}u}{\partial {x}^{3}}+\frac{{l}^{2}}{{R}^{2}}\frac{{\partial }^{3}u}{\partial x\partial {\theta }^{2}})+\frac{{B}_{12}}{R}(\frac{\partial {\psi }_{x}}{\partial x}+{l}^{2}\frac{{\partial }^{3}{\psi }_{x}}{\partial {x}^{3}}+\frac{{l}^{2}}{{R}^{2}}\frac{{\partial }^{3}{\psi }_{x}}{\partial x\partial {\theta }^{2}})\\ +\frac{{A}_{22}}{R}(\,-\,\frac{1}{R}\frac{\partial v}{\partial \theta }-\frac{w}{R}+\frac{{l}^{2}}{{R}^{2}}\frac{{\partial }^{3}v}{\partial {x}^{2}\partial \theta }+\frac{{l}^{2}}{{R}^{2}}\frac{{\partial }^{2}w}{\partial {x}^{2}}+\frac{{l}^{2}}{{R}^{3}}\frac{{\partial }^{3}v}{\partial {\theta }^{3}}+\frac{{l}^{2}}{{R}^{3}}\frac{{\partial }^{2}w}{\partial {\theta }^{2}})\\ +\frac{{B}_{22}}{R}(\,-\,\frac{1}{R}\frac{\partial {\psi }_{\theta }}{\partial \theta }+\frac{{l}^{2}}{{R}^{2}}\frac{{\partial }^{3}{\psi }_{\theta }}{\partial {x}^{2}\partial \theta }+\frac{{l}^{2}}{{R}^{3}}\frac{{\partial }^{3}{\psi }_{\theta }}{\partial {\theta }^{3}})\\ +{K}_{s}{A}_{55}(\frac{\partial {\psi }_{x}}{\partial x}-\frac{{\partial }^{2}w}{\partial {x}^{2}}-{l}^{2}\frac{{\partial }^{3}{\psi }_{x}}{\partial {x}^{3}}-{l}^{2}\frac{{\partial }^{4}w}{\partial {x}^{4}}-\frac{{l}^{2}}{{R}^{2}}\frac{{\partial }^{3}{\psi }_{x}}{\partial x\partial {\theta }^{2}}-\frac{{l}^{2}}{{R}^{2}}\frac{{\partial }^{4}w}{\partial {x}^{2}\partial {\theta }^{2}})\\ +\frac{{K}_{s}{A}_{44}}{R}(\frac{\partial {\psi }_{\theta }}{\partial \theta }-\frac{1}{R}\frac{{\partial }^{2}w}{\partial {\theta }^{2}}-\frac{1}{R}\frac{\partial v}{\partial \theta }-{l}^{2}\frac{{\partial }^{3}{\psi }_{\theta }}{\partial {x}^{2}\partial \theta }-\frac{{l}^{2}}{R}\frac{{\partial }^{4}w}{\partial {x}^{2}\partial {\theta }^{2}}+\frac{{l}^{2}}{R}\frac{{\partial }^{3}v}{\partial {x}^{2}\partial \theta }\\ -\frac{{l}^{2}}{{R}^{2}}\frac{{\partial }^{3}{\psi }_{\theta }}{\partial {\theta }^{3}}-\frac{{l}^{2}}{{R}^{3}}\frac{{\partial }^{4}w}{\partial {\theta }^{4}}+\frac{{l}^{2}}{{R}^{3}}\frac{{\partial }^{3}v}{\partial {\theta }^{3}})=(1-{\mu }^{2}{\nabla }^{2})\left({I}_{0}(\frac{{\partial }^{2}w}{\partial {t}^{2}})\right)\end{array}$$

6 $$\begin{array}{cc} & \delta {\psi }_{x}:{B}_{11}(\frac{{\partial }^{2}u}{\partial {x}^{2}}-{l}^{2}\frac{{\partial }^{4}u}{\partial {x}^{4}}-\frac{{l}^{2}}{{R}^{2}}\frac{{\partial }^{4}u}{\partial {x}^{2}\partial {\theta }^{2}})+{D}_{11}(\frac{{\partial }^{2}{\psi }_{x}}{\partial {x}^{2}}-{l}^{2}\frac{{\partial }^{4}{\psi }_{x}}{\partial {x}^{4}}-\frac{{l}^{2}}{{R}^{2}}\frac{{\partial }^{4}{\psi }_{x}}{\partial {x}^{2}\partial {\theta }^{2}})\\  & +{B}_{12}(\frac{1}{R}\frac{{\partial }^{2}v}{\partial x\partial \theta }+\frac{1}{R}\frac{\partial w}{\partial x}-\frac{{l}^{2}}{{R}^{2}}\frac{{\partial }^{4}v}{\partial {x}^{3}\partial \theta }-\frac{{l}^{2}}{{R}^{2}}\frac{{\partial }^{3}w}{\partial {x}^{3}}-\frac{{l}^{2}}{{R}^{3}}\frac{{\partial }^{4}v}{\partial x\partial {\theta }^{3}}-\frac{{l}^{2}}{{R}^{3}}\frac{{\partial }^{3}w}{\partial x\partial {\theta }^{2}})\\  & +{D}_{12}(\frac{1}{R}\frac{{\partial }^{2}{\psi }_{\theta }}{\partial x\partial \theta }+\frac{{l}^{2}}{{R}^{2}}\frac{{\partial }^{4}{\psi }_{\theta }}{\partial {x}^{3}\partial \theta }-\frac{{l}^{2}}{{R}^{3}}\frac{{\partial }^{4}{\psi }_{\theta }}{\partial x\partial {\theta }^{3}})\\  & +\frac{{B}_{66}}{R}(\frac{1}{R}\frac{{\partial }^{2}u}{\partial {\theta }^{2}}+\frac{{\partial }^{2}v}{\partial x\partial \theta }-\frac{{l}^{2}}{R}\frac{{\partial }^{4}u}{\partial {x}^{2}\partial {\theta }^{2}}-{l}^{2}\frac{{\partial }^{4}v}{\partial {x}^{3}\partial \theta }-\frac{{l}^{2}}{{R}^{3}}\frac{{\partial }^{4}u}{\partial {\theta }^{4}}-\frac{{l}^{2}}{{R}^{2}}\frac{{\partial }^{4}v}{\partial x\partial {\theta }^{3}})\\  & +\frac{{D}_{66}}{R}(\frac{1}{R}\frac{{\partial }^{2}{\psi }_{x}}{\partial {\theta }^{2}}+\frac{{\partial }^{2}{\psi }_{\theta }}{\partial x\partial \theta }-\frac{{l}^{2}}{R}\frac{{\partial }^{4}{\psi }_{x}}{\partial {x}^{2}\partial {\theta }^{2}}-{l}^{2}\frac{{\partial }^{4}{\psi }_{\theta }}{\partial {x}^{3}\partial \theta }-\frac{{l}^{2}}{{R}^{3}}\frac{{\partial }^{4}{\psi }_{x}}{\partial {\theta }^{4}}-\frac{{l}^{2}}{{R}^{2}}\frac{{\partial }^{4}{\psi }_{\theta }}{\partial x\partial {\theta }^{3}})\\  & -{K}_{s}{A}_{55}({\psi }_{x}+\frac{\partial w}{\partial x}-{l}^{2}\frac{{\partial }^{2}{\psi }_{x}}{\partial {x}^{2}}-{l}^{2}\frac{{\partial }^{3}w}{\partial {x}^{3}}-\frac{{l}^{2}}{{R}^{2}}\frac{{\partial }^{2}{\psi }_{x}}{\partial {\theta }^{2}}-\frac{{l}^{2}}{{R}^{2}}\frac{{\partial }^{3}w}{\partial x\partial {\theta }^{2}})\\ = & (1-{\mu }^{2}{\nabla }^{2})({I}_{1}\frac{{\partial }^{2}u}{\partial {t}^{2}}+{I}_{2}\frac{{\partial }^{2}{\psi }_{x}}{\partial {t}^{2}})\end{array}$$

7 $$\begin{array}{c}\delta {\psi }_{\theta }:\frac{{B}_{12}}{R}(\frac{{\partial }^{2}u}{\partial x\partial \theta }-{l}^{2}\frac{{\partial }^{4}u}{\partial {x}^{3}\partial \theta }-\frac{{l}^{2}}{{R}^{2}}\frac{{\partial }^{4}u}{\partial x\partial {\theta }^{3}})+\frac{{D}_{12}}{R}(\frac{{\partial }^{2}{\psi }_{x}}{\partial x\partial \theta }-{l}^{2}\frac{{\partial }^{4}{\psi }_{x}}{\partial {x}^{3}\partial \theta }-\frac{{l}^{2}}{{R}^{2}}\frac{{\partial }^{4}{\psi }_{x}}{\partial x\partial {\theta }^{3}})\\ +\frac{{B}_{22}}{R}(\frac{1}{R}\frac{{\partial }^{2}v}{\partial {\theta }^{2}}-\frac{1}{R}\frac{\partial w}{\partial \theta }-\frac{{l}^{2}}{{R}^{2}}\frac{{\partial }^{4}v}{\partial {x}^{2}\partial {\theta }^{2}}-\frac{{l}^{2}}{{R}^{2}}\frac{{\partial }^{3}w}{\partial {x}^{2}\partial \theta }-\frac{{l}^{2}}{{R}^{3}}\frac{{\partial }^{4}v}{\partial {\theta }^{4}}-\frac{{l}^{2}}{{R}^{3}}\frac{{\partial }^{3}w}{\partial {\theta }^{3}})\\ +\frac{{D}_{22}}{R}(\frac{1}{R}\frac{{\partial }^{2}{\psi }_{\theta }}{\partial {\theta }^{2}}-\frac{{l}^{2}}{R}\frac{{\partial }^{4}{\psi }_{\theta }}{\partial {x}^{2}\partial {\theta }^{2}}-\frac{{l}^{2}}{{R}^{3}}\frac{{\partial }^{4}{\psi }_{\theta }}{\partial {\theta }^{4}})\\ +{B}_{66}(\frac{1}{R}\frac{{\partial }^{2}u}{\partial x\partial \theta }-\frac{{\partial }^{2}v}{\partial {x}^{2}}-\frac{{l}^{2}}{R}\frac{{\partial }^{4}u}{\partial {x}^{3}\partial \theta }-{l}^{2}\frac{{\partial }^{4}v}{\partial {x}^{4}}-\frac{{l}^{2}}{{R}^{3}}\frac{{\partial }^{4}u}{\partial x\partial {\theta }^{3}}-\frac{{l}^{2}}{{R}^{2}}\frac{{\partial }^{4}v}{\partial {x}^{2}\partial {\theta }^{2}})\\ +{D}_{66}(\frac{1}{R}\frac{{\partial }^{2}{\psi }_{x}}{\partial x\partial \theta }-\frac{{\partial }^{2}{\psi }_{\theta }}{\partial {x}^{2}}-\frac{{l}^{2}}{R}\frac{{\partial }^{4}{\psi }_{x}}{\partial {x}^{3}\partial \theta }-{l}^{2}\frac{{\partial }^{4}{\psi }_{\theta }}{\partial {x}^{4}}-\frac{{l}^{2}}{{R}^{3}}\frac{{\partial }^{4}{\psi }_{x}}{\partial x\partial {\theta }^{3}}-\frac{{l}^{2}}{{R}^{2}}\frac{{\partial }^{4}{\psi }_{\theta }}{\partial {x}^{2}\partial {\theta }^{2}})\\ -{k}_{s}{A}_{44}({\psi }_{\theta }+\frac{1}{R}\frac{\partial w}{\partial \theta }-\frac{v}{R}-{l}^{2}\frac{{\partial }^{2}{\psi }_{\theta }}{\partial {x}^{2}}-\frac{{l}^{2}}{R}\frac{{\partial }^{3}w}{\partial {x}^{2}\partial \theta }+\frac{{l}^{2}}{R}\frac{{\partial }^{2}v}{\partial {x}^{2}}-\frac{{l}^{2}}{{R}^{2}}\frac{{\partial }^{2}{\psi }_{\theta }}{\partial {\theta }^{2}}-\frac{{l}^{2}}{{R}^{3}}\frac{{\partial }^{3}w}{\partial {\theta }^{3}}\\ +\frac{{l}^{2}}{{R}^{3}}\frac{{\partial }^{2}v}{\partial {\theta }^{2}})=(1-{\mu }^{2}{\nabla }^{2})\left[{I}_{1}\left(\frac{{\partial }^{2}v}{\partial {t}^{2}}\right)+{I}_{2}\left(\frac{{\partial }^{2}{\psi }_{\theta }}{\partial {t}^{2}}\right)\right]\end{array}$$

here, the defined elements in Eqs. (3)–(7) are explained as:

8 $$\begin{array}{c}\{\begin{array}{cccccc}{A}_{11} & {A}_{12} & {A}_{22} & {A}_{66} & {A}_{44} & {A}_{55}\end{array}\}={\int }_{-h/2}^{h/2}\{\begin{array}{cccccc}{C}_{11} & {C}_{12} & {C}_{22} & {C}_{66} & {C}_{44} & {C}_{55}\end{array}\}dz\\ \{\begin{array}{cccc}{B}_{11} & {B}_{12} & {B}_{22} & {B}_{66}\end{array}\}={\int }_{-h/2}^{h/2}\{\begin{array}{cccc}{C}_{11} & {C}_{12} & {C}_{22} & {C}_{66}\end{array}\}zdz\\ \{\begin{array}{cccc}{D}_{11} & {D}_{12} & {D}_{22} & {D}_{66}\end{array}\}={\int }_{-h/2}^{h/2}\{\begin{array}{cccc}{C}_{11} & {C}_{12} & {C}_{22} & {C}_{66}\end{array}\}{z}^{2}dz\\ \{\begin{array}{ccc}{I}_{0} & {I}_{1} & {I}_{2}\end{array}\}={\int }_{-h/2}^{h/2}\rho (z,T)\{\begin{array}{ccc}1 & z & {z}^{2}\end{array}\}dz\end{array}$$
