# Author Correction: Dynamic response of the nonlocal strain-stress gradient in laminated polymer composites microtubes

**DOI:** 10.1038/s41598-020-74969-y

**Published:** 2020-10-30

**Authors:** Mohammad Amin Oyarhossein, As’ad Alizadeh, Mostafa Habibi, Mahmoud Makkiabadi, Mohsen Daman, Hamed Safarpour, Dong Won Jung

**Affiliations:** 1 Department of Civil Engineering, University of Aveiro, Aveiro, Portugal; 2 Department of Mechanical Engineering, Urmia University of Technology, Urmia, Iran; 3 Center of Excellence in Design, Robotics, and Automation, School of Mechanical Engineering, Sharif University of Technology, Tehran, Iran; 4 Department of Mechanical Engineering, Amirkabir University of Technology, Tehran, Iran; 5 Department of Mechanics, Imam Khomeini International University, Qazvin, Iran; 6 School of Mechanical Engineering, Jeju National University, Jeju, Jeju-do 690-756 South Korea; 7 Department of Mechanical Engineering, College of Engineering, University of Zakho, Zakho, Iraq

Correction to: *Scientific Reports* 10.1038/s41598-020-61855-w, published online 27 March 2020

The original version of this Article omitted an affiliation for As’ad Alizadeh. The correct affiliations for As’ad Alizadeh are listed below:

Department of Mechanical Engineering, Urmia University of Technology, Urmia, Iran

Department of Mechanical Engineering, College of Engineering, University of Zakho, Zakho, Iraq

This has now been corrected in the HTML and PDF versions of this Article.

